# Deploying Machine Learning Techniques for Human Emotion Detection

**DOI:** 10.1155/2022/8032673

**Published:** 2022-02-02

**Authors:** Ali I. Siam, Naglaa F. Soliman, Abeer D. Algarni, Fathi E. Abd El-Samie, Ahmed Sedik

**Affiliations:** ^1^Department of Embedded Network Systems Technology, Faculty of Artificial Intelligence, Kafrelsheikh University, Kafr El-Sheikh, Egypt; ^2^Department of Information Technology, College of Computer and Information Sciences, Princess Nourah Bint Abdulrahman University, Riyadh 84428, Saudi Arabia; ^3^Department of the Robotics and Intelligent Machines, Faculty of Artificial Intelligence, Kafrelsheikh University, Kafr El-Sheikh, Egypt

## Abstract

Emotion recognition is one of the trending research fields. It is involved in several applications. Its most interesting applications include robotic vision and interactive robotic communication. Human emotions can be detected using both speech and visual modalities. Facial expressions can be considered as ideal means for detecting the persons' emotions. This paper presents a real-time approach for implementing emotion detection and deploying it in the robotic vision applications. The proposed approach consists of four phases: preprocessing, key point generation, key point selection and angular encoding, and classification. The main idea is to generate key points using MediaPipe face mesh algorithm, which is based on real-time deep learning. In addition, the generated key points are encoded using a sequence of carefully designed mesh generator and angular encoding modules. Furthermore, feature decomposition is performed using Principal Component Analysis (PCA). This phase is deployed to enhance the accuracy of emotion detection. Finally, the decomposed features are enrolled into a Machine Learning (ML) technique that depends on a Support Vector Machine (SVM), k-Nearest Neighbor (KNN), Naïve Bayes (NB), Logistic Regression (LR), or Random Forest (RF) classifier. Moreover, we deploy a Multilayer Perceptron (MLP) as an efficient deep neural network technique. The presented techniques are evaluated on different datasets with different evaluation metrics. The simulation results reveal that they achieve a superior performance with a human emotion detection accuracy of 97%, which ensures superiority among the efforts in this field.

## 1. Introduction

Recognition of human emotions is a vital phase, which is involved in several applications such as augmented and virtual reality [1, 2], advanced driver assistance systems [3], human computer interaction [4], and security systems [5–7]. Humans have several ways of interpreting the emotions of others, such as speech and linguistic aspects [8] and facial expressions [9–11]. Furthermore, emotions can be detected based on gaze direction [12] and biosignals including electroencephalogram (EEG) and electrocardiogram (ECG). Emotional expressions are used for intelligent Human-Robot Interaction (HRI). Emotion analysis can also be used to track the students' emotions to enhance the learning environment. Therefore, the students can learn better using this approach. Such information obtained through emotion analysis is useful in monitoring of the overall mood of a group of persons to identify any destructive events [13]. In human interaction, 7% of the affective information is conveyed by words, 38% is conveyed by speech tone, and 55% is conveyed by facial expressions [14]. Therefore, the facial emotion analysis can be a dependable approach to recognize human emotions for HRI applications.

The robot vision issue can be handled using thermal images [15–17] and RGB images [18]. This paper presents a real-time study for emotion detection and deployment in robotic vision applications. The proposed approach consists of four phases: preprocessing, feature extraction and selection, feature decomposition, and classification. Feature extraction and selection is carried out by MediaPipe face mesh algorithm. This algorithm is based on real-time deep learning. In addition, the feature decomposition phase is performed by PCA. This phase is deployed to enhance the accuracy of emotion detection. It is required to decompose the extracted features using the Singular Value Decomposition (SVD). Finally, the obtained features are enrolled into a selected classifier. In addition, an MLP deep neural network is utilized. The introduced techniques are assessed on different datasets with the help of different evaluation metrics. Moreover, this paper introduces a hardware implementation of the proposed models. The main contributions of this work can be summarized as follows:

1. A novel fast and robust emotion detection framework for robotic vision applications is proposed.
2. Emotion face mesh is introduced depending on automatic key point determination from face images.
3. Key point angular encoding is presented to generate sensitive and distinguishable angular features.
4. Emotion classification is performed depending on various machine learning techniques.
5. A brief comparison is made between the deployed techniques in terms of accuracy, scalability, and processing time.

The remaining parts of this paper are organized as follows.  Section 2 covers the works introduced in the literature.  Section 3 shows the datasets utilized in this work. Furthermore, the proposed methodology is discussed in Section 4, and its simulation results are given in Section 5. Moreover, the result discussion highlights the performance of the proposed approach among the works in the literature in Section 6. Finally, the paper concluding remarks are given in Section 6.

## 2. Related Work

Several researchers presented their frameworks to handle the issue of HRI. The work in [19] offers a conditional-generative-adversarial-network-based (CGAN-based) framework to reduce intraclass variances by managing facial expressions individually, while simultaneously learning generative and discriminative representations. A generator *G* and three discriminators make up this architecture (Di, Da, and Dexp). Any query face image is transformed into a prototypic facial expression form with certain factors kept by the generator *G*. An accuracy of 81.83% was achieved. A model based on CNN was proposed in the work of [20]. It was designed for smile detection, emotion recognition, and gender classification. Therefore, it is considered as a multi-task model. It achieved an accuracy of 71.03%.

Some efforts have been presented for emotion detection using deep learning. The work in [21] introduced a deep CNN to deploy a facial expression recognition system. This system can automatically extract the features of facial expressions to allow automatic recognition. In addition, it consists of input, preprocessing, recognition, and output modules. Furthermore, it was used to simulate and assess the recognition performance under the effect of several aspects such as network structure, learning rate, and preprocessing on both the Japanese Female Facial Expression (JAFFE) dataset and the Extended Cohn–Kanade (CK+) dataset. To make the results more convincing, the authors used the k-Nearest Neighbor (KNN) technique. For JAFFE and CK+ datasets, the performance accuracies are 76.7442% and 80.303%, respectively. Another model was proposed in [22]. It was tested on a facial expression dataset of HDR images, considering a collection of faces under different lighting conditions. It is based on SVM, Local Binary Patterns (LBPs), and appearance. It works depending on the Speeded-Up Robust Feature (SURF) transform to conduct the emotion recognition task. This model revealed accuracy levels up to 80%. In [23], the authors presented a model for submission to the fifth Emotion Recognition in the Wild (EmotiW 2017) group-level emotion recognition subchallenge. They deployed a CNN to extract features from the detected face images. Another role for the CNN is to be trained for the face identification task, rather than traditional pretraining on emotion recognition problems. In the final pipeline, an ensemble of Random Forest (RF) classifiers was learned to predict an emotion score using an available training set. This model achieved an accuracy of 75.4% on the validation data.

Another trend in this field is to detect emotions from videos. The authors of [24] presented a hybrid deep learning model for emotion detection from videos. A spatial CNN is used for processing of static facial images and a temporal CNN for optical flow images. These two processing branches are used to learn high-level spatial and temporal features on video segments, separately. These two CNNs are fine-tuned using pretrained CNN models and target video facial expression datasets. A deep fusion network, which is deployed using a Deep Belief Network (DBN) model, fuses the collected features from the segment-level spatial and temporal branches. The obtained fused features are enrolled into a linear SVM for facial expression classification tasks. The authors achieved an accuracy of 75.39%. Moreover, another video-based emotion detection algorithm was presented in [25]. The authors investigated different ways for pooling spatial and temporal data. For video-based face expression identification, they discovered that pooling spatial and temporal information together is more efficient. Unlike the framework given in [24], this work is end-to-end trainable for whole-video recognition. The goal of this framework is to create a trainable deep neural network framework for pattern identification that integrates spatial and temporal information from video using CNNs and LSTMs. This framework achieved an accuracy of 65.72%.

## 3. Dataset Description

The proposed models are evaluated on three datasets: Cohn–Kanade (CK+) [26], Japanese Female Facial Expression (JAFFE) [27], and Real-world Affective Faces Database (RAF-DB) [28]. A description of each of them is given below.

### 3.1. Cohn–Kanade [CK+]

**Figure 1:**
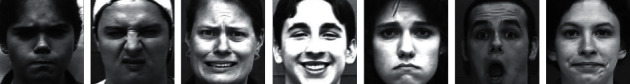
Examples of images from CK+ dataset.

The CK+ dataset [26] consists of 593 video sequences from 123 participants. Each sequence contains images beginning from onset (neutral frame) and progressing to the peak expression (last frame). The label associated with each sequence is depicted from the peak expression. The dataset contains images for seven different expressions: anger, contempt, fear, disgust, happiness, surprise, and sadness. The images have a resolution of 640 × 480 pixels. In this work, the images are cropped into 48 × 48 pixels to focus on the subject face.  Figure 1 shows sample images for each expression.

### 3.2. Japanese Female Facial Expression (JAFFE)

**Figure 2:**
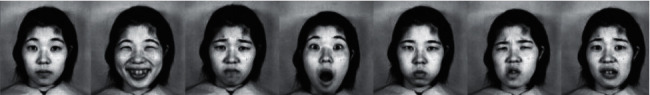
Examples of images from JAFFE dataset.

The JAFFE dataset [27] has 213 photos of ten different female actors posing for seven different facial expressions. There are six primary expressions: happiness, sadness, surprise, anger, disgust, and fear, plus one neutral expression. The images have a resolution of 256 × 256 pixels.  Figure 2 shows sample images for each expression.

### 3.3. Real-World Affective Face Database (RAF-DB)

**Figure 3:**
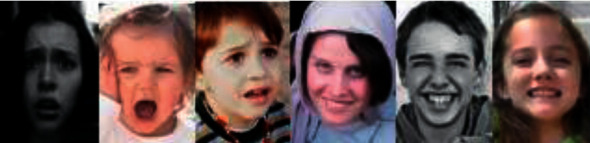
Examples of images from RAF-DB.

RAF-DB [28] contains 15,339 facial images with uncontrolled poses and illumination from thousands of individuals of different ages and races. The images within the RAF-DB are labeled by approximately 40 annotators. The database includes six basic expressions plus a neutral expression. Sample images from RAF-DB are shown in Figure 3.

## 4. Proposed Methodology

**Figure 4:**
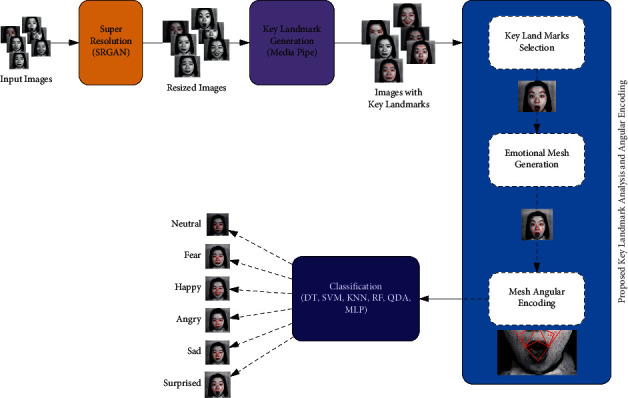
The proposed framework.

This paper presents an emotion detection approach based on deep and machine learning techniques. The main idea of this approach is to deploy deep learning as an automatic key point generator using MediaPipe technique. Hence, a sensitive mathematical process is performed to encode the generated key points into a set of distinguishable features. In addition, different machine learning techniques are implemented on the extracted features to perform the classification task. The proposed approach consists of four main phases. The first phase is image preprocessing in which a super-resolution task is carried out using SRGAN. In the second phase, we deploy MediaPipe to generate key landmarks on the face images. Furthermore, we present a key landmark analysis and an angular encoding module. This module contains three subphases (key landmark selection, emotional mesh generation, and mesh angular encoding). The main idea of this module is to generate an emotional mesh that connects the selected key landmarks. Hence, the obtained mesh is encoded into angular values to generate a feature map. Moreover, the generated feature map is enrolled into a classifier to be discriminated into six categories.  Figure 4 represents the proposed framework.

### 4.1. Preprocessing

Generally, the images that are captured by robotic vision devices have a limited resolution due to the hardware limitations of cameras involved in such systems. Furthermore, most of the available datasets for human emotion recognition are down-sized because of the storage limitations. Therefore, the first module in the proposed approach is the super-resolution. In addition, the proposed approach involves angular feature extraction from the geometry of the face images, which requires a clarified representation of the landmarks and boundaries of the face images to allow proper facial emotion recognition. SRGAN [29], a Generative Adversarial Network (GAN) for image Super-Resolution (SR), is employed in the current research to increase the perceptual quality of images prior to further processes. With SRGAN, the images are super-resolved with a 4*x* upscaling factor, while minimizing the Mean SquareError (MSE) between the super-resolved and original images and maximizing the Peak Signal-to-Noise Ratio (PSNR).

**Figure 5:**
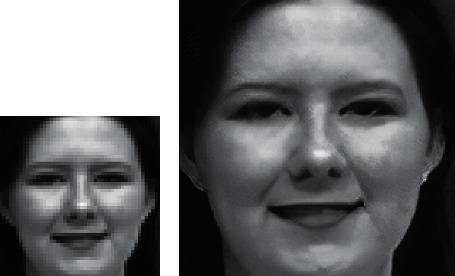
Preprocessing step (super-resolution): (a) original image [48 × 48]; (b) 4*x* upscaled super-resolved image using SRGAN [192 × 192].

Figure 5 illustrates the preprocessing step by employing the SRGAN. The figure displays an original image selected from the CK+ dataset and the corresponding super-resolved image after SRGAN. The original image size is 48 × 48 pixels, and the super-resolved image size is 192 × 192 pixels.

### 4.2. Key Landmark Generation

**Figure 6:**
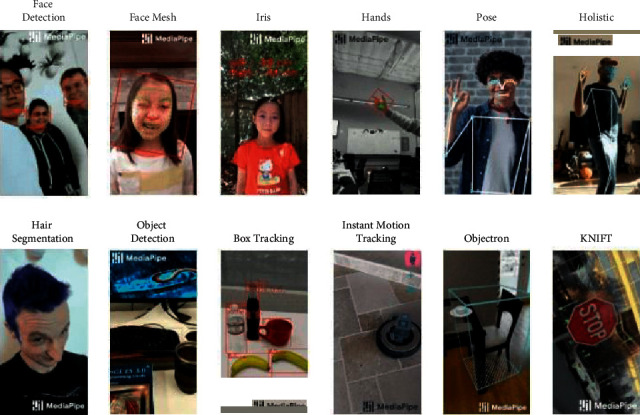
Different examples of ML solutions in MediaPipe [30].

The process of key landmark generation is performed using deep MediaPipe technique. MediaPipe [30] is an open-source ML framework developed by Google and devoted to building real-life computer vision applications. MediaPipe capabilities allow developers to focus on algorithm or model development, while using MediaPipe to iteratively improve their application with results that are consistent across different devices and platforms [31]. Solutions that are currently implemented with MediaPipe include face detection, face mesh annotation, iris localization, hand detection, pose estimation, hair segmentation, object detection and tracking, and 3D object detection (Objectron). These solutions are released in different platforms: mobile (Android and iOS), C++, Python, and JS. Real-life examples of ML solutions in MediaPipe are shown in Figure 6.

**Figure 7:**
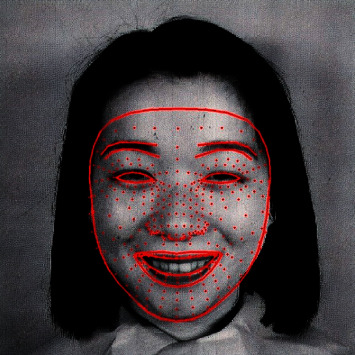
An image with 468 annotated landmarks using MediaPipe face mesh.

In the current work, the face mesh solution from the MediaPipe framework is employed to annotate the landmarks and boundaries of the face. Face mesh calculates 468 3D face landmarks in real time. It uses ML to infer 3D surface geometry using just a single camera input without a specialized depth sensor [32]. The solution provides a real-time performance, even on mobile devices.  Figure 7 displays an image selected from the JAFFE dataset with the 468 facial landmarks annotated on the image.

### 4.3. Proposed Key Landmark Analysis and Angular Encoding

This paper presents a key landmark analysis and an angular encoding module. This module contains three subphases (key landmark selection, emotional face mesh generation, and mesh angular encoding). The main idea of this module is to generate an emotional mesh, which connects the selected key landmarks. Hence, the obtained mesh is encoded into angular values to generate a feature map. In the following subsections, a discussion for each step in this module is presented.

#### 4.3.1. Key Landmark Selection

**Figure 8:**
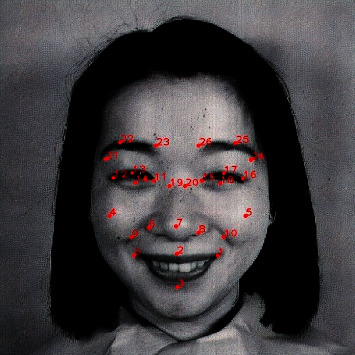
The 27 key landmarks and their locations.

As discussed earlier, the MediaPipe face mesh solution provides face detection capability and 468 facial landmarks spread over the face, along with their locations (*x* and *y* coordinates for each detected landmark). In the proposed model, only 27 key landmarks are selected from the 468 detected landmarks. These key landmarks are used later to define the vertices of the emotion face mesh.  Table 1 describes the selected key landmarks and the corresponding MediaPipe landmark IDs. The 27 key landmarks and their locations on a test face image are shown in Figure 8.

The selection of the key landmarks and their locations is based on the Facial Action Coding System (FACS) [33, 34], which encodes movements of individual facial muscles. It can be used to describe facial actions that make up an expression based on changes in facial muscles regardless of emotion. The movement of particular facial muscles, known as Action Units (AUs), is encoded by FACS. This requires unique instantaneous changes in facial appearance [35].  Table 2 describes the facial emotion-related AUs and the corresponding FACS names. A graphic-based demonstration for FACS with isolated AUs is illustrated in [36]. Hence, facial emotions can be represented using reliable combinations of different AUs, as demonstrated in Table 3. Each key landmark location is chosen such that it is more probably affected by a specific emotion-related AU, which seeks better recognition of facial expressions.

#### 4.3.2. Emotional Mesh Generation

**Figure 9:**
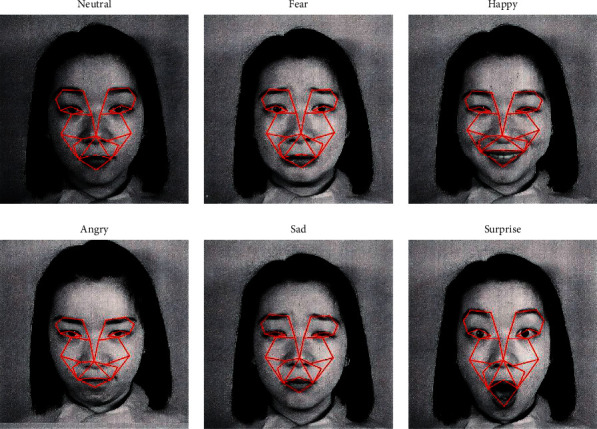
Samples of emotion face mesh for different emotions.

After selection of the key landmarks, emotion face mesh is created, consisting of 27 vertices inferred from the selected key landmarks. Edges of the emotion face mesh, which define the connections between vertices, are drawn to establish a closed mesh structure.  Table 4 defines the edges that constitute the emotion face mesh, as well as the start and end vertices for each edge. The vertices IDs are defined in Table 1. The mesh yields 27 vertices and 38 edges. Deformation of emotion face mesh measured by the deviation of angles between edges reflects facial muscle contraction and relaxation, which will be used to identify facial emotions.  Figure 9 displays the emotion face mesh for sample images selected from the JAFFE dataset with different emotions.

#### 4.3.3. Mesh Angular Encoding

**Figure 10:**
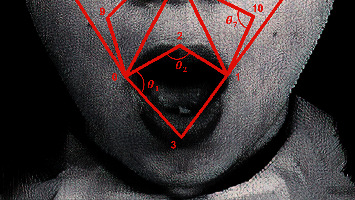
An example showing the features (*θ*_1_, *θ*_2_, and *θ*_7_) and their locations.

After acquiring the key landmarks and establishing the emotion face mesh, we use the mesh to extract the relevant features for emotion classification. The relevant features employed are geometric features, since most emotions can be detected from geometric changes. Ten features are extracted, defining angles between specific edges of the emotion face mesh. The angles are represented in degrees in the range of (0°, 360°). These features are then fed to the ML classifiers to learn from them to identify each emotion. The low dimensionality of features (10 features) makes them more resistant to local facial changes. In addition, the classifiers can be trained in a much shorter time. Moreover, the overall complexity of the proposed framework is significantly reduced. The list of angles taken as discriminant features for emotion classification, and the three vertices IDs forming each angle are given in Table 5. An example depicting the angular features and their locations on a test face image is shown in Figure 10.

**Figure 11:**
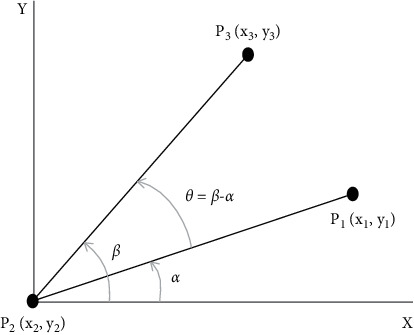
Computing the angle between two lines (edges).

The angle between the three vertices can be computed as follows (consider Figure 11).

The angle *θ* between the line (edge) connecting *P*_2_ and *P*_3_ and the line (edge) connecting the points *P*_2_ and *P*_1_ is unknown.

The angle *β* between the line *P*_2_-*P*_3_ and the *X*-axis can be computed as

(1) $$\begin{array}{c} \beta =\;\tan^{-1}\left(\frac{y_{3}-y_{2}}{x_{3}-x_{2}}\right). \end{array}$$

Similarly, the angle *α* between the line *P*_2_-*P*_1_ and the *X*-axis can be computed as

(2) $$\begin{array}{c} \alpha =\;\tan^{-1}\left(\frac{y_{1}-y_{2}}{x_{1}-x_{2}}\right). \end{array}$$

Hence, the angle *θ* will be

(3) $$\begin{array}{c} \theta =\beta -\alpha =\;\tan^{-1}\left(\frac{y_{3}-y_{2}}{x_{3}-x_{2}}\right)-\;\tan^{-1}\left(\frac{y_{1}-y_{2}}{x_{1}-x_{2}}\right). \end{array}$$

Using the above procedure, ten angles between prescribed edges in the emotion face mesh are computed, and then used for classification. Angle values are all positive, where negative values can be avoided by adding 360° to the values. Furthermore, the generated feature maps are redistributed using PCA to enhance their distribution.

### 4.4. Classification

In this work, we develop an automated facial expression identifier to recognize human emotions for robotic vision applications. Discriminant features extracted from a face (Section 4.3) are fed to classifiers to recognize the emotion in the given face. DT, KNN, a multiclass SVM [37], Gaussian NB, MLP with backpropagation, QDA, RF, and LR classifiers are used for classification. The trial-and-error method and grid-search [38] are conducted to identify the optimal structure and hyperparameters of classifiers. In addition, 10-fold cross-validation is employed to estimate the optimal hyperparameter combinations to avoid overfitting. The optimal hyperparameters of classifiers adopted in the current work are investigated in Table 6.

**Figure 12:**
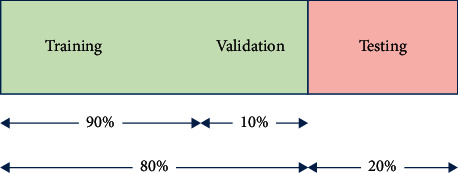
Pipeline for fragmentation of datasets.

The images in the dataset are divided into two parts: training part and testing part. The training part is used to train/validate the classifier, and the testing part is used to test the performance of the classifier. The splitting scheme is 80/20, as shown in Figure 12. The 10-fold cross-validation adopted in the current model employs further splitting of the training part into ten folds (subsets). After that, nine folds are used to train the classifier, while the remaining fold is used to validate the training. This process continues until each of the ten folds is used exactly once for validation. The optimal configurations identified in the training stage are then applied in the testing stage.

## 5. Experimental Results

Experiments are performed on an Intel Core i3 machine with 8 GB RAM. Python 3.9 is used as the development environment. The OpenCV 4.5 and SRGAN libraries are used for image preprocessing. MediaPipe 0.8.6 library is used as the building block for the key feature extraction. Scikit-learn 0.24.2 [39] is used for implementing the machine learning classifiers and computing the evaluation metrics for the proposed model. NumPy, Pandas, Math, OS, and Matplotlib are used as supplementary libraries. The accuracy, precision, recall, *F*1-score, and training time are the five metrics used to evaluate the proposed framework. The training time is recorded based on the average of five runs. The proposed model is evaluated using two different datasets: CK+ (6 classes) and JAFFE (6 classes), which are benchmark datasets for facial expression classification. For CK+, 784 images are used for training, while 197 images are used for classification. For JAFFE, the training set contains 164 images, and the testing set contains 42 images.

To evaluate the performance of the proposed model, eight classifiers are employed to classify facial expressions across two benchmark datasets. The hyperparameters employed for each classifier are presented in Table 6. The classification is based on ten features extracted from images in each dataset using the procedure described in Section 4.

**Figure 13:**
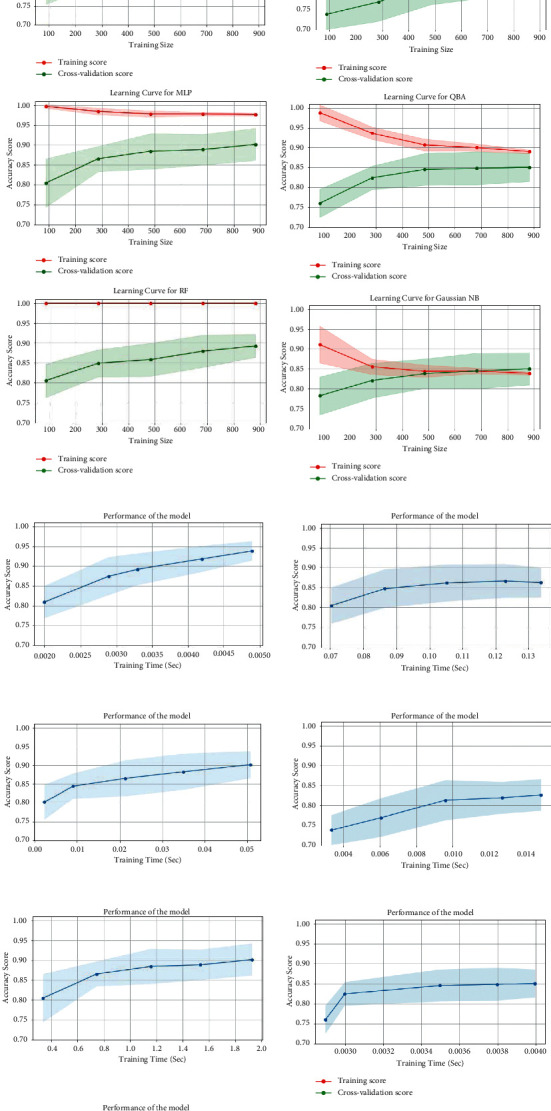
The accuracy (a) and performance vs. training time (b) for different classifiers (KNN, SVM, MLP, RF, LR, DT, QDA, and Gaussian NB) for facial expression recognition using the proposed model on CK+ dataset.

Learning curves, which determine cross-validation scores and behaviors for different training sizes for the adopted classifiers in case of CK+, are shown in Figure 13.

**Figure 14:**
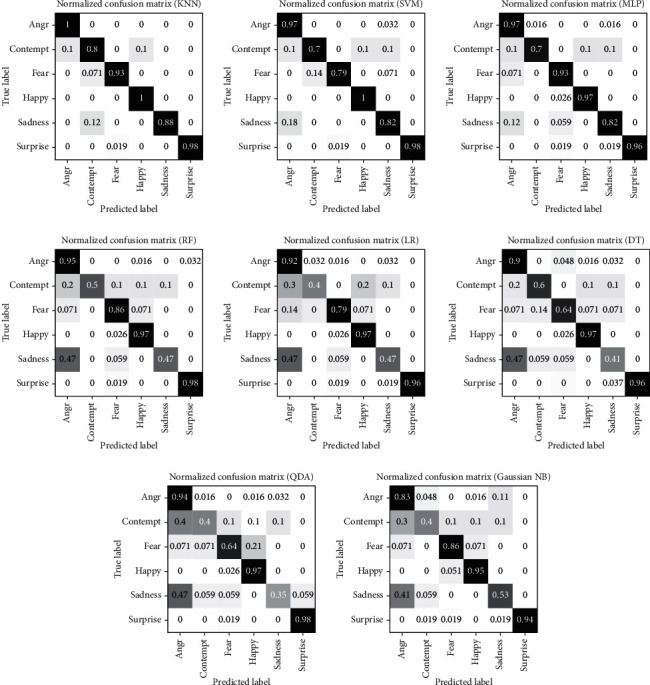
Confusion matrices of emotion detection classifiers on CK+ dataset.

**Figure 15:**
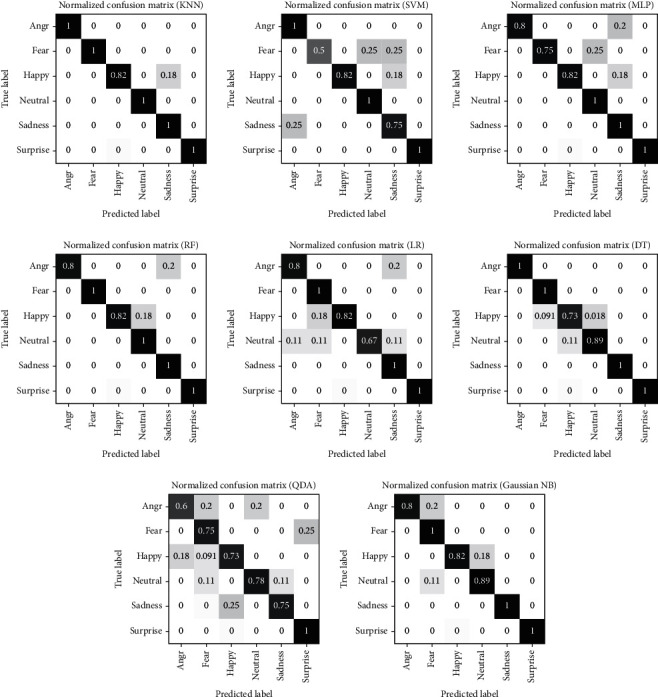
Confusion matrices of emotion detection classifiers on JAFFE dataset.

The confusion matrix for each classifier on the CK+ dataset using the proposed model is shown in Figure 14. It shows that the per-class accuracies of Anger, Happy, and Surprise classes have higher values with all classifiers than those of other emotions, while the Contempt and Sadness classes have lower per-class accuracies. Moreover, the confusion matrices for classifiers on JAFFE dataset are shown in Figure 15.

**Figure 16:**
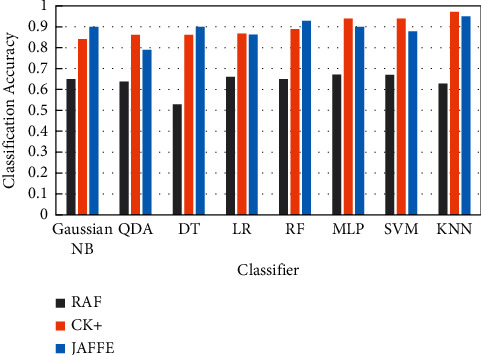
Accuracies of eight ML classifiers on RAF, CK+, and JAFFE datasets for facial expression classification using the proposed approach.

The performances of the proposed framework with eight classifiers on CK+, JAFFE, and RAF-DB [28] datasets are presented in Tables 7–9. The illustrated results show the classification report including accuracy, precision, recall, and *F*1-score as well as the training time taken for each classifier. A visual comparison between the classifier accuracies across the used datasets is shown in Figure 16.

Results reveal that the KNN classifier outperforms other classifiers in terms of accuracy, precision, recall, and *F*1-score. It achieved the best accuracies of 97% and 95% on CK+ and JAFFE datasets, respectively. The accuracies for Gaussian NB, QDA, DT, LR, RF, MLP, and SVM classifers on the CK+ are 84%, 86%, 86%, 87%, 89%, 94%, and 94%, respectively, and those on the JAFFE are 90%, 79%, 90%, 86%, 93%, 90%, and 88%, respectively. In addition, the time required to train the KNN and Gaussian NB is 0.005 sec on CK+. It is the lowest time compared to those of other classifiers. The MLP and RF classifiers have the highest training times, which are 1.82 sec and 0.74 sec, respectively. Moreover, the proposed models are evaluated on theRAF-DB. The results of this evaluation reveal that the proposed MLP and SVM models can be considered as good emotion detection models for this database, especially with an accuracy of 67% for both models. Therefore, the proposed approach provides a variety of models, which are optimal for robust emotion detection environments.

## 6. Discussion

The simulation results reveal that the proposed approach shows a high performance in human emotion detection. Furthermore, they clarify that the proposed encoding module has a superior performance with the deployed classifiers including KNN, SVM, and MLP. In this section, a brief comparison is presented between the proposed approach and the works in the literature as illustrated in Table 10. It can be observed that the proposed approach has a superior performance among the efforts in this field.

## 7. Conclusion

The issue of Human-Robot Interaction (HRI) has been discussed in this paper. As a solution, the paper presented a novel approach for facial expression recognition. This proposed approach consists of four phases, which are carried out to extract key points from facial images using a real-time algorithm (MediaPipe). Furthermore, these key points are enrolled into a sequence of selection, mesh generator, and angular encoding modules. Moreover, the generated feature maps are classified using several classification algorithms, including SVM, KNN, RF, QDA, NB, LR, DT, and MLP. The novelty of the proposed approach is highlighted in the proposed key point analysis and angular encoding algorithm. This algorithm is efficient, because it generates only ten features (angular values), which are discriminative for different emotional classification categories. The proposed approach has been evaluated on CK+, JAFEE, and RAF-DB datasets. It reveals a superior performance in terms of accuracy of detection and processing time evaluation metrics. Furthermore, the low dimensionality of extracted features enables the ML-based approaches to reach an optimum performance in a short time with much lower computational cost than those of the DL-based approaches, which require more time for convergence and need much computational cost.

In addition, the future work that can be deduced from this paper is introducing a method for emotion detection from other modalities such as videos, spoken words, and written text. Furthermore, hardware implementation of the proposed approach is a research trend, which we are working on. Moreover, further machine learning techniques such as dictionary learning and semi-supervised learning can be performed to solve this issue.

## Figures and Tables

**Table 1:** Selected key landmarks (vertices) and the corresponding MediaPipe landmarks.

Key landmark ID	MediaPipe landmark ID	Description
0	61	Mouth end (right)
1	292	Mouth end (left)
2	0	Upper lip (middle)
3	17	Lower lip (middle)
4	50	Right cheek
5	280	Left cheek
6	48	Nose right end
7	4	Nose tip
8	289	Nose left end
9	206	Upper jaw (right)
10	426	Upper jaw (left)
11	133	Right eye (inner)
12	130	Right eye (outer)
13	159	Right upper eyelid (middle)
14	145	Right lower eyelid (middle)
15	362	Left eye (inner)
16	359	Left eye (outer)
17	386	Left upper eyelid (middle)
18	374	Left lower eyelid (middle)
19	122	Nose bridge (right)
20	351	Nose bridge (left)
21	46	Right eyebrow (outer)
22	105	Right eyebrow (middle)
23	107	Right eyebrow (inner)
24	276	Left eyebrow (outer)
25	334	Left eyebrow (middle)
26	336	Left eyebrow (inner)

**Table 2:** List of AU codes and FACS descriptors.

AU	FACS name
1	Inner brow raiser
2	Outer brow raiser
4	Brow lowerer
5	Upper lid raiser
6	Cheek raiser
7	Lid tightener
8	Lips toward each other
9	Nose wrinkler
10	Upper lip raiser
11	Nasolabial deepener
12	Lip corner puller
13	Sharp lip puller
14	Dimpler
15	Lip corner depressor
16	Lower lip depressor
17	Chin raiser
18	Lip pucker
19	Tongue show
20	Lip stretcher
21	Neck tightener
22	Lip funneler
23	Lip tightener
24	Lip pressor
25	Lips part
26	Jaw drop
27	Mouth stretch
28	Lip suck
29	Jaw thrust
30	Jaw sideways
31	Jaw clencher

**Table 3:** Emotion description in terms of AUs.

Emotion	AU
Happiness	6 + 12
Sadness	1 + 4 + 15
Surprise	1 + 2 + 5 + 26
Fear	1 + 2 + 4 + 5+7 + 20 + 26
Anger	4 + 5 + 7 + 23
Disgust	9 + 15 + 17
Contempt	12 + 14

**Table 4:** Edges and vertices of the emotion face mesh.

Edge	Connected vertices IDs
1	(0, 2)
2	(0, 3)
3	(1, 2)
4	(1, 3)
5	(7, 6)
6	(7, 8)
7	(6, 4)
8	(8, 5)
9	(6, 9)
10	(9, 0)
11	(4, 0)
12	(8, 10)
13	(10, 1)
15	(7, 19)
16	(7, 20)
17	(7, 0)
18	(7, 1)
19	(19, 23)
20	(19, 14)
21	(23, 22)
22	(22, 21)
23	(21, 12)
24	(12, 13)
25	(12, 14)
26	(11, 13)
27	(11, 14)
28	(14, 4)
29	(20, 26)
30	(26, 25)
31	(25, 24)
32	(24, 16)
33	(16, 17)
34	(16, 18)
35	(15, 17)
36	(15, 18)
37	(18, 20)
38	(18, 5)

**Table 5:** List of features (angles) and the three enclosing vertices.

Features	Enclosing vertices IDs
*θ*_1_	(2, 0,3)
*θ*_2_	(0, 2,1)
*θ*_3_	(6, 7, 8)
*θ*_4_	(9, 7, 10)
*θ*_5_	(0, 7, 1)
*θ*_6_	(1, 5, 8)
*θ*_7_	(1, 10, 8)
*θ*_8_	(13, 12, 14)
*θ*_9_	(21, 22, 23)
*θ*_10_	(6, 19, 23)

**Table 6:** List of hyperparameters of adopted classifiers.

Classifier	Hyperparameters
DT	Criterion: “gini”
	min_samples_leaf: 1
	min_samples_split: 2
	ccp_alpha: 0

KNN	n_neighbors:1
	leaf_size: 30
	Metric: “minkowski”
	p: 2
	Weights: “uniform”

SVM	C: 275
	Gamma: “scale”
	Kernel: “rbf”

Gaussian NB	var_smoothing: 1e-09

MLP	Num_hidden_layers:2
	hidden_layer_sizes: [28, 28]
	Activation: “relu”
	max_iter: 200
	Solver: “adam”

QDA	tol = 0.0001

RF	n_estimators:79
	Criterion: “entropy”

LR	Solver: “lbfgs”
	C: 1.0
	fit_intercept: true

**Table 7:** Comparison of eight ML classifiers on CK+ dataset.

Classifier	Classification accuracy	Precision	Recall	*F*1-score	Training time (seconds)
Gaussian NB	0.84	0.84	0.84	0.84	0.005
QDA	0.86	0.85	0.86	0.85	0.006
DT	0.86	0.85	0.86	0.85	0.018
LR	0.87	0.86	0.87	0.86	0.15
RF	0.89	0.90	0.89	0.88	0.74
MLP	0.94	0.94	0.94	0.94	1.82
SVM	0.94	0.94	0.94	0.94	0.06
KNN	0.97	0.97	0.97	0.97	0.005

**Table 8:** Comparison of eight ML classifiers on JAFFE dataset.

Classifier	Classification accuracy	Precision	Recall	*F*1-score	Training time (seconds)
Gaussian NB	0.90	0.93	0.90	0.91	0.005
QDA	0.79	0.80	0.79	0.79	0.005
DT	0.90	0.91	0.90	0.90	0.005
LR	0.86	0.90	0.86	0.86	0.09
RF	0.93	0.94	0.93	0.93	0.33
MLP	0.90	0.94	0.90	0.91	0.41
SVM	0.88	0.91	0.88	0.88	0.008
KNN	0.95	0.97	0.95	0.95	0.002

**Table 9:** Comparison of eight ML classifiers on RAF-DB.

Classifier	Classification accuracy	Precision	Recall	*F*1-score	Training time (seconds)
Gaussian NB	0.65	0.62	0.65	0.61	0.01
QDA	0.64	0.62	0.64	0.61	0.02
DT	0.53	0.52	0.53	0.52	0.26
LR	0.66	0.62	0.66	0.63	0.9
RF	0.65	0.61	0.65	0.62	11
MLP	0.67	0.64	0.67	0.64	20
SVM	0.67	0.64	0.67	0.64	24.5
KNN	0.63	0.60	0.63	0.61	0.053

**Table 10:** Comparison between the proposed work and the state-of-the-art works.

Work	Year	Method	Accuracy (%)			Time (s)
			JAFFE	CK+	RAF	
[40]	2018	CNN	50.12	93.64		—
[28]	2019	mSVM	88.95	91.98	65.12	—
		LDA	83.45	92.33	56.93	—
[41]	2018	RF	—	93.4	—	—
[42]	2018	SVM	—	95.8	—	—
[43]	2018	AlexNet	93	90.2	—	—
		VGG16	96	92.4	—	0.94
[44]	2018	VGG19	93	93	—	—
[45]	2021	CNN	96.8	86.5	—	62.5
[46]	2018	AlexNet	—	—	55.6	—
		VGG	—	—	58.2	—

**Proposed**	**2021**	**MLP**	**90**	**94**	**67**	**1.12**
		**SVM**	**88**	**94**	**67**	**0.034**
		**KNN**	**95**	**97**	**63**	**0.004**
		**LR**	**86**	**87**	**66**	**0.12**

## Data Availability

The data used to support the findings of this study are available from the corresponding author upon request.
